# A gene expression atlas of *Nicotiana tabacum* across various tissues at transcript resolution

**DOI:** 10.3389/fpls.2025.1500654

**Published:** 2025-02-07

**Authors:** Shizhou Yu, Jufen Wan, Tenghang Xu, Jie Zhang, Linggai Cao, Jie Liu, Hongfeng Liu, Xueliang Ren, Zhixiao Yang

**Affiliations:** ^1^ Molecular Genetics Key Laboratory of China Tobacco, Guizhou Academy of Tobacco Science, Guiyang, China; ^2^ State Key Laboratory for Crop Stress Resistance and High-Efficiency Production, Northwest A&F University, Yangling, China; ^3^ College of Agronomy and Biotechnology, Hebei Normal University of Science and Technology, Qinhuangdao, China; ^4^ Guiyang Branch Company of Guizhou Tobacco Company, Guiyang, China

**Keywords:** *Nicotiana tabacum*, ASE, tissue-specific transcript, stress response, SQTL

## Abstract

Alternative splicing (AS) expands the transcriptome diversity by selectively splicing exons and introns from pre-mRNAs to generate different protein isoforms. This mechanism is widespread in eukaryotes and plays a crucial role in development, environmental adaptation, and stress resistance. In this study, we collected 599 tobacco RNA-seq datasets from 35 projects. 207,689 transcripts were identified in this study, of which 35,519 were annotated in the reference genome, while 172,170 transcripts were newly annotated. Additionally, tissue-specific analysis revealed 4,585 transcripts that were uniquely expressed in different tissues, highlighting the complexity and specialization of tobacco gene expression. The analysis of AS events (ASEs) across different tissues showed significant variability in the expression levels of ASE-derived transcripts, with some of these transcripts being associated with stress resistance, such as the *geranyl diphosphate synthase* (*GGPPS*). Moreover, we identified 21,763 splicing quantitative trait locus (sQTLs), which were enriched in genes involved in biological processes such as histone acetylation. Furthermore, sQTLs involved genes related to plant hormone signal transduction, terpenoid backbone biosynthesis, and other resistance pathways. These findings not only reveal the diversity of gene expression in tobacco but also provide new insights and strategies for improving tobacco quality and resistance.

## Introduction

AS is a crucial mechanism in gene expression regulation, where selective splicing of exons and introns in pre-mRNA generates multiple distinct mRNA isoforms, thereby expanding the coding capacity of the genome. This process is widespread among eukaryotes and plays a key role in human diseases, plant and animal development, and stress responses. Studies have shown that various gene mutations affecting the global regulation of AS or altering AS of specific genes are associated with human brain diseases (Licatalosi and Darnell, 2006; Raj and Blencowe, 2015). Comparative analysis of AS in adipose tissue from different sheep breeds revealed that genes related to ASE are closely related to adipose tissue development (Miao et al., 2023). Temperature changes regulate intron retention and affect starch accumulation in *Arabidopsis* (Seo et al., 2011). Numerous studies indicate that AS plays a significant role in stress responses. Analysis of AS in *Arabidopsis* under normal physiological conditions and various abiotic stress treatments revealed that isoforms generated by the AS play roles in responding to abiotic stress (Filichkin et al., 2010). Significant changes in AS were observed in wheat under stress conditions such as high temperature and drought (Liu et al., 2018). The *OsCYP19-4* gene produces various transcripts through AS, which have different subcellular localizations, and these isoforms play distinct roles in responding to cold stress (Lee et al., 2016).

With the advancement of AS research, it has become evident that genetic variations also play an integral role in regulating AS patterns. sQTL is genetic variation sites associated with ASE that influence the composition of gene expression isoforms by regulating pre-mRNA splicing patterns (Dwivedi et al., 2023). For instance, sQTL in different *Arabidopsis* ecotypes are enriched in genes related to circadian rhythm, flowering, and stress responses (Khokhar et al., 2019), indicating that single nucleotide polymorphisms (SNPs) regulate gene expression by altering transcript isoforms. The identification of sQTL in barley (*Hordeum vulgare*) core accessions under Cd exposure has revealed potential mechanisms for Cd accumulation in plants (Deng et al., 2022). sQTL studies provide new insights into the complexity of gene regulation, uncovering how genetic variation influences splicing mechanisms to regulate gene expression and ultimately affect biological phenotypes.

Tobacco (*Nicotiana tabacum*) is a widely cultivated plant with significant economic and agricultural importance, serving as the primary source of tobacco used in the global cigarette industry (Warner, 2000). Beyond its economic value, tobacco has also become a model plant in research due to its well-characterized genome and ease of genetic manipulation (Ganapathi et al., 2004). However, like many other crops, tobacco is affected by various environmental stresses, including drought, salinity, extreme temperatures, and pathogen attacks, which can severely impact its growth, yield, and quality. Understanding the mechanisms by which tobacco responds to these abiotic and biotic stresses is crucial for developing more resilient varieties.

Currently, research on AS is primarily focused on crops like *Arabidopsis* and rice. However, the regulatory mechanisms and functional significance of AS in tobacco. A deeper understanding of AS mechanisms not only helps to uncover the diversity of gene expression but also provides new insights and strategies for crop improvement. In this study, we performed transcriptomic analysis based on RNA-seq expression profiles of 599 tobacco samples from 13 tissues, including transcript identification, tissue-specific transcripts identification, expression characteristics analysis of transcripts produced by different ASEs, and sQTL identification. These findings greatly enrich the tobacco transcriptome library and offer valuable insights for tobacco breeding.

## Materials and methods

### Data sources

RNA-seq data was retrieved from 35 BioProjects available in NCBI, covering 599 samples across 13 tissues (**Supplementary Table S1**). Tobacco genome data was obtained from the Sol Genomics Network database (https://solgenomics.net/), specifically using the genome version published by Edwards et al. in 2017 (Edwards et al., 2017).

### Transcript assembly, processing and expression

The SRA Toolkit (https://github.com/ncbi/sra-tools/wiki) was used with default parameters to convert SRA files into FASTQ format. The FASTQ data underwent quality control using fastp (Chen et al., 2018) with default settings, resulting in 599 clean datasets. Post-QC FASTQ files were aligned to the tobacco genome using STAR (v2.7.10a) (Dobin et al., 2013) with default parameters. Transcript assembly and merging were performed with StringTie (v2.1.7) (Pertea et al., 2015) using the ‘-merge’ option on the aligned data. GffCompare (0.11.2) (Pertea and Pertea, 2020) was employed to compare the merged transcript assemblies with the reference genome annotations. Transcript quantification was conducted using RSEM (Li and Dewey, 2011) with default parameters. Transcripts not matched to genes or those aligned in the opposite direction to gene transcription were considered potential non-coding RNAs and excluded from further analysis. Additionally, transcripts with coordinates overlapping two or more genes were identified as fusion transcripts and excluded from subsequent analysis to ensure the accuracy of the results. Gene quantification was carried out using featureCounts (Liao et al., 2014) with default settings. tSNE clustering analysis of transcript expression levels was performed using the R package Rtsne (Krijthe and van der Maaten, 2015), and GO, KEGG, and PFAM enrichment analyses were conducted using the clusterProfiler v4.0 package (Wu et al., 2021).

### Tissue-specific expressed transcripts

Following the methodology of Kryuchkova-Mostacci et al (Kryuchkova-Mostacci and Robinson-Rechavi, 2017), Tau index (τ) was used as a measure of tissue specificity for each transcript (Yanai et al., 2005). In brief, we first calculated the median TPM (Transcripts Per Million) for each transcript across all tissues. Transcripts with a median TPM below 0.1 were considered not expressed (TPM = 0). The corrected TPM values were then log2-transformed using the formula log2(TPM + 1). For each transcript, only those with a sum of log2(TPM + 1) across all tissues greater than 0.1 were retained; all other transcripts were discarded. The filtered results were subsequently used to calculate Tau (García-Pérez et al., 2021). The calculation formula is:


$$\tau =\frac{\sum_{i=1}^{n}(1-\frac{x_{i}}{\underset{1\leq i\leq n}{\max}x_{i}})}{n-1}$$

n represents the total number of tissues, and Xi denotes the expression level of transcript X in tissue i. Transcripts with a Tau value greater than 0.85 and a maximum expression level exceeding 5 TPM were considered tissue-specific. Based on the correspondence between transcripts and genes, GO enrichment analysis was performed on the host genes of these tissue-specific transcripts. Host genes are those from which the corresponding transcripts are transcribed.

### Correlation between transcripts and host genes

we calculated the Pearson correlation coefficient between the expression levels of transcripts and their host genes. Under the condition that the p-value is less than 0.05, a Pearson correlation coefficient greater than 0.3 was considered indicative of a positive correlation between transcript and gene expression, while a coefficient less than -0.3 was considered indicative of a negative correlation. Otherwise, the expression of the transcript was deemed uncorrelated with that of its host gene (Wu et al., 2023).

### AS analysis

SUPPA2 (v2.3) (Trincado et al., 2018) was used with default parameters to identify alternative splicing events (ASEs), followed by filtering of the identified events. Briefly, we first inferred ASEs based on the transcript structure and expression levels of genes using the “–generateEvents” parameter. Then, the Percent Spliced In (PSI) value for each ASE in each sample was calculated using the “–psiPerEvent” parameter. We retained events with a PSI value greater than 0.1 in at least 5% of the samples (n = 14) to generate a set of high-confidence events.

### Tobacco leaves SNP calling

This study conducted SNP calling for 375 tobacco leaf samples. The specific steps were as follows: First, BAM files generated from the mapping of tobacco leaf samples to the reference genome were sorted using SAMtools (v1.21) (Li et al., 2009). Next, PCR duplicates were marked using the MarkDuplicates function of Picard (https://github.com/broadinstitute/picard). Subsequently, the SplitNCigarReads function in GATKwas applied to split reads into exon segments (removing Ns while maintaining grouping information) and hard-clip any sequences overhanging into intronic regions (Van Auwera and O’Connor, 2020). SNP calling was then performed with HaplotypeCaller function of GATK. Detected SNPs were filtered using the VariantFiltration tool of GATK, retaining only those with a Fisher Strand (FS) < 30.0 and a Quality by Depth (QD) > 2.0. Further filtering was conducted with SelectVariants tool of GATK to retain only biallelic variants. Finally, VCFtools (v0.1.16) (Danecek et al., 2011) was used to filter the SNP dataset with parameters set to –max-missing 0.9 and –maf 0.05, retaining SNP sites with a missing rate below 10% and a minor allele frequency above 5% in the population.

### Identification of sQTL

ASEs were identified using LeafCutter (Li et al., 2018), and the official script prepare_phenotype_table.py was used to clusters the identified introns, filters out introns present in less than 40% of the population or those with minimal variation, and normalizes the PSI values. We employed PEER (v1.0) to infer hidden determinants from the normalized PSI values, selecting the top 10 PEER factors as covariates for sQTL analysis to correct for batch effects and other influencing factors. Additionally, we used the top five PCA results from the prepare_phenotype_table.py output as covariates. Principal component analysis (PCA) was performed using PLINK (v1.90b6.21) (Purcell et al., 2007), and the top 10 principal components were selected to correct for population structure. The PEER factors, PSI PCA results, and VCF PCA results were combined to create a covariate file for sQTL identification. The normalized PSI value for each intron splicing event was used as the phenotype, and sQTLs were identified using FastQTL with the ‘-normal’ parameter. sQTLs located within 100 kb of an ASE were defined as cis-sQTLs. An FDR threshold of <0.05 was applied to identify all significant cis-sQTLs.

## Results

### Extensive transcriptome diversity and complexity in tobacco

**Figure 1:**
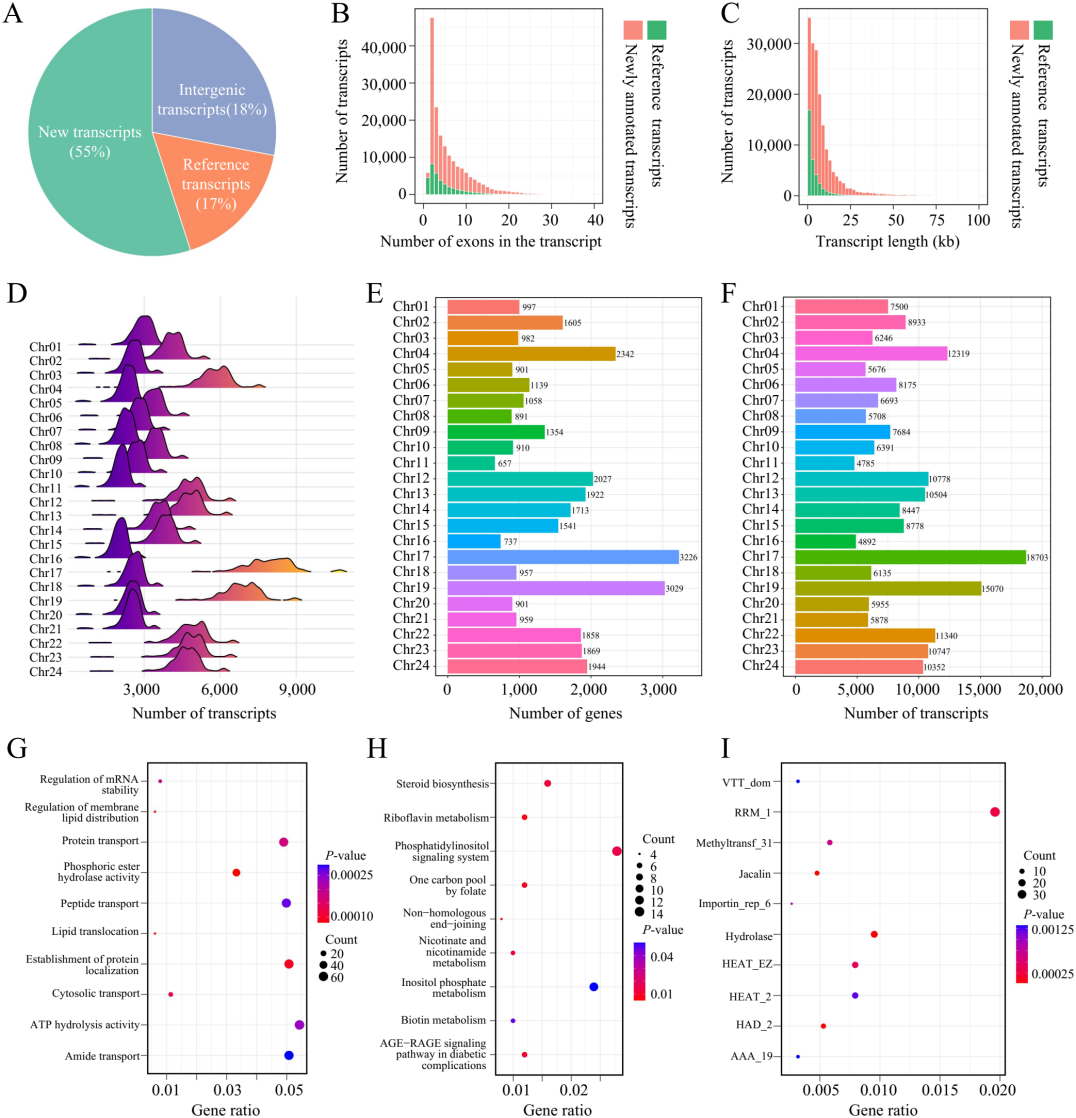
Transcriptome profiling and functional analysis in tobacco. **(A)** Transcripts identified in 599 tobacco RNA-seq datasets. "New Transcripts," "Reference Transcripts," and "Intergenic Transcripts" correspond to newly annotated transcripts, transcripts annotated in the reference and transcripts that are located in intergenic regions, respectively. **(B)** Histogram of exon counts per transcript. **(C)** Histogram of transcript length distribution. **(D)** Distribution of expressed transcripts across chromosomes in different samples. **(E)** Gene counts across different chromosomes. **(F)** Gene counts across different chromosomes. **(G)** GO enrichment analysis of the top 5% genes with the highest isoform counts. **(H)** KEGG enrichment analysis of the top 5% genes with the highest isoform counts. **(I)** PFAM enrichment analysis of the top 5% genes with the highest isoform counts.

In this study, a total of 207,689 transcripts were identified from 599 transcriptome datasets derived from 13 different tissues. Based on whether these transcripts were annotated in the reference genome, they were classified into newly annotated transcripts and those already annotated in the reference genome. A total of 172,170 newly annotated transcripts were identified, accounting for 83% of all transcripts. This indicates that the tobacco transcriptome contains a large number of transcripts that were not annotated in the reference genome, demonstrating the complexity and diversity of tobacco gene expression. According to the reference genome’s GFF file, 113,553 transcripts overlap with known genes, while 58,617 transcripts are located in intergenic regions. These transcripts were considered potential non-coding RNAs and were excluded from downstream analysis (**Figure 1A**). In terms of exon count, the number of transcripts decreases as the number of exons increases. Most transcripts have fewer than 20 exons, with transcripts containing 2 exons being the most common, totaling over 50,000. Compared to the annotated transcripts in the reference genome, the newly annotated transcripts include more transcripts with over 20 exons (**Figure 1B**). Regarding transcript length distribution, the number of transcripts shows a negative correlation with length. The majority of transcripts are shorter than 25 kb, while transcripts longer than 25 kb are mostly newly annotated (**Figure 1C**). These results suggest that the newly annotated transcripts not only dominate in quantity but also exhibit more complex structural characteristics, such as longer lengths and more exons. The transcriptional intensity across the population varies among different chromosomes. The number of expressed transcripts across the 599 samples typically clusters within a peak range for each chromosome. For example, chromosomes 17 and 19 of tobacco have the highest number of expressed transcripts, ranging between 6,000 to 8,000. Chr02, Chr12, Chr13, Chr14, Chr15, Chr21, Chr22, and Chr23 have a number of expressed transcripts ranging between 3,000 to 6,000, while the remaining chromosomes generally have fewer than 3,000 expressed transcripts (**Figure 1D**). The differences in the number of expressed transcripts across chromosomes are related to the number of genes annotated on each chromosome. Generally, the more genes annotated on a chromosome, the more transcripts are expressed. However, although chromosome Chr06 has fewer annotated genes than Chr09, Chr06 shows more expressed transcripts than Chr09 (**Figures 1E, F**), which may be due to differences in transcriptional regulation on Chr06. These findings underscore the complexity of the tobacco transcriptome and provide new insights into the spatial distribution of gene expression across chromosomes.

To explore the relationship between the number of transcript isoforms and gene function, we performed functional enrichment analysis on the top 5% of genes with the highest isoform numbers. The results showed that genes with a large number of transcript isoforms play key roles in important biological processes, such as the regulation of mRNA stability, regulation of membrane lipid distribution, phosphoric ester hydrolase activity, and protein transport (**Figure 1G**). The transcript diversity of these genes may enable cells to flexibly adjust functions under different environmental conditions, thus adapting to physiological demands. Additionally, KEGG pathway analysis revealed that these genes are enriched in critical pathways such as the phosphatidylinositol signaling system, inositol phosphate metabolism, and steroid biosynthesis (**Figure 1H**), further suggesting that these genes play essential roles in maintaining cellular metabolic balance and signal regulation. PFAM analysis showed that these genes are enriched in protein families such as RRM_1, Methyltransf_31, and Hydrolase (**Figure 1I**), indicating that transcript diversity may contribute to gene expression regulation through mechanisms involving RNA processing, metabolic regulation, and material transport. Overall, these findings reveal that genes with a large number of transcript isoforms potentially have important functions in various biological processes, providing new insights into the relationship between gene diversity and gene function.

### Transcript-specific expression patterns across different tissues

**Figure 2:**
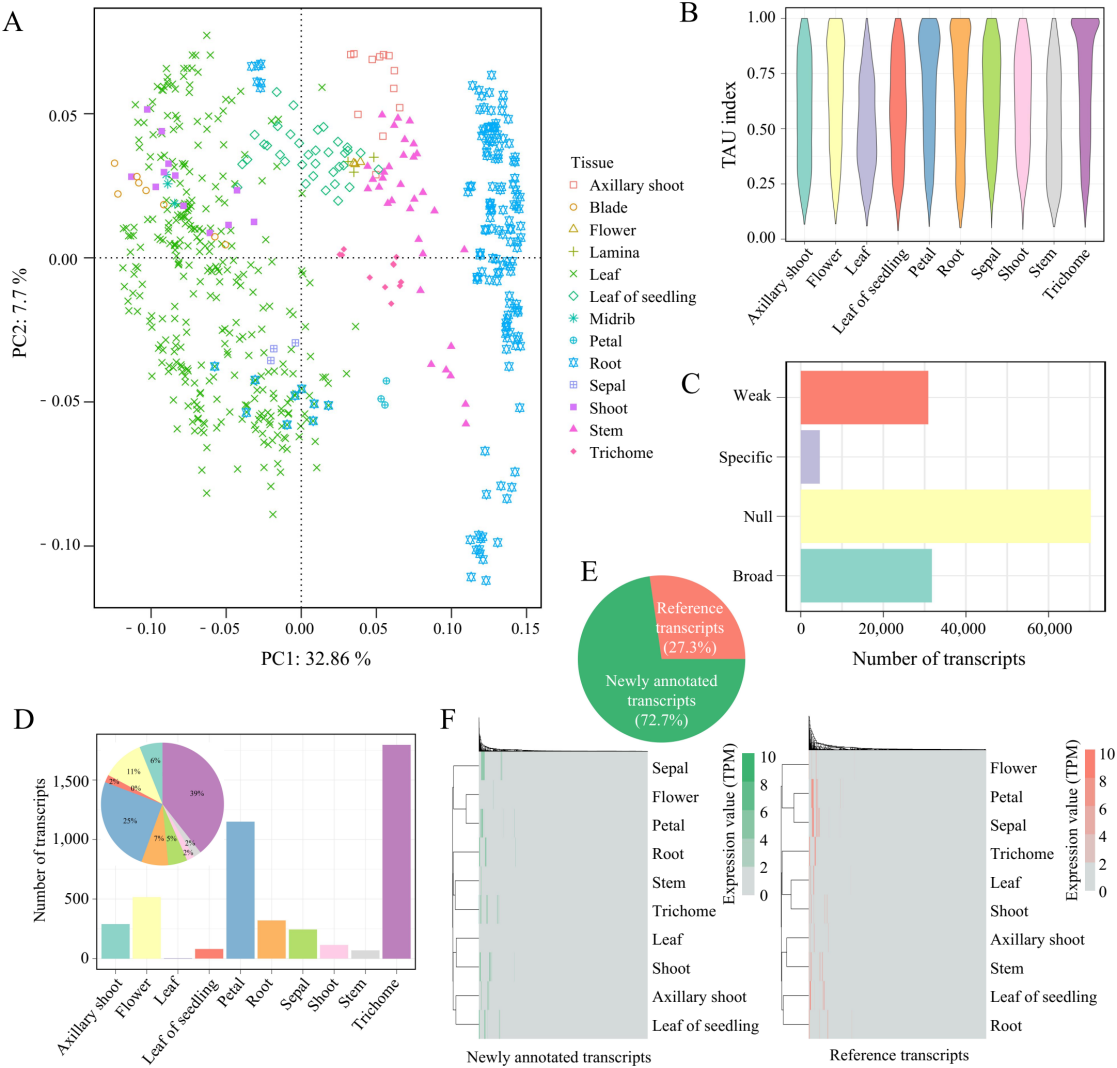
Tissue-specific transcripts across different tissues. **(A)** tSNE clustering of transcripts. **(B)** Distribution of Tau for transcripts across different tissues. **(C)** Transcripts number of different transcript types. **(D)** Number and proportion of tissue-specific transcripts in different tissues. **(E)** Proportion of tissue-specific transcripts in transcripts annotated in the reference and newly annotated transcripts. **(F)** Expression clustering of tissue-specific transcripts in transcripts annotated in the reference (reference transcripts) and newly annotated transcripts. Each column represents the expression level of different transcripts in the samples.

The distinct functions of plant tissues lead to tissue-specific gene expression patterns (Shi et al., 2021). However, AS can cause certain transcripts to be expressed specifically in certain tissues. To identify the tissue-specific transcripts across 10 tobacco issues, we first excluded non-coding transcripts and conducted tSNE dimensionality reduction clustering on 137,568 transcripts. The results showed significant differences in transcript expression between different tissues. Notably, several tobacco leaf tissues (leaf, blade, midrib, shoot) exhibited low variability, clustering together in the tSNE plot, except for the leaf of seedlings (**Figure 2A**). Therefore, we combined blade, lamina, and midrib with leaf for tissue-specific analysis. We also compared the distribution of Tau values across tissues (**Figure 2B**), finding that trichomes showed a peak near 1, indicating a higher number of tissue-specific transcripts in this tissue. Subsequently, we categorized transcripts into four classes based on their expression levels (null, weak, broad, and tissue-specific, **Supplementary Table S2**). The results showed that more than half (51%) were low-expressed null transcripts (TPM <1 in all tissues; n=70,211), 22.4% were weak transcripts (TPM <5 in all tissues; n=30,868), and 23.1% were broad transcripts (TPM >=5, Tau<0.85; n=31,763), with only 3.5% (4,585) being tissue-specific transcripts (TPM >=5, Tau >=0.85; n=4,585) (**Figure 2C**). Among these, trichomes had the highest number of tissue-specific transcripts (**Figure 2D**). Notably, 27.3% (1,251) of these tissue-specific transcripts were derived from the reference genome annotation, while newly annotated transcripts accounted for 72.7% (3,334), highlighting the significance of AS-derived novel transcripts in tissue-specific analysis (**Figure 2E**). Furthermore, expression clustering analysis of these transcripts revealed similar expression patterns between the transcripts annotated in the reference and newly annotated transcripts (**Figure 2F**). This study used tSNE clustering to uncover transcript expression differences between different tobacco tissues, identifying numerous tissue-specific transcripts, particularly in trichomes, and demonstrating the importance of novel transcript assembly for a deeper understanding of tissue-specific expression.

### Tissue-specific transcripts showed inconsistent expression patterns with tissue-specific genes

**Figure 3:**
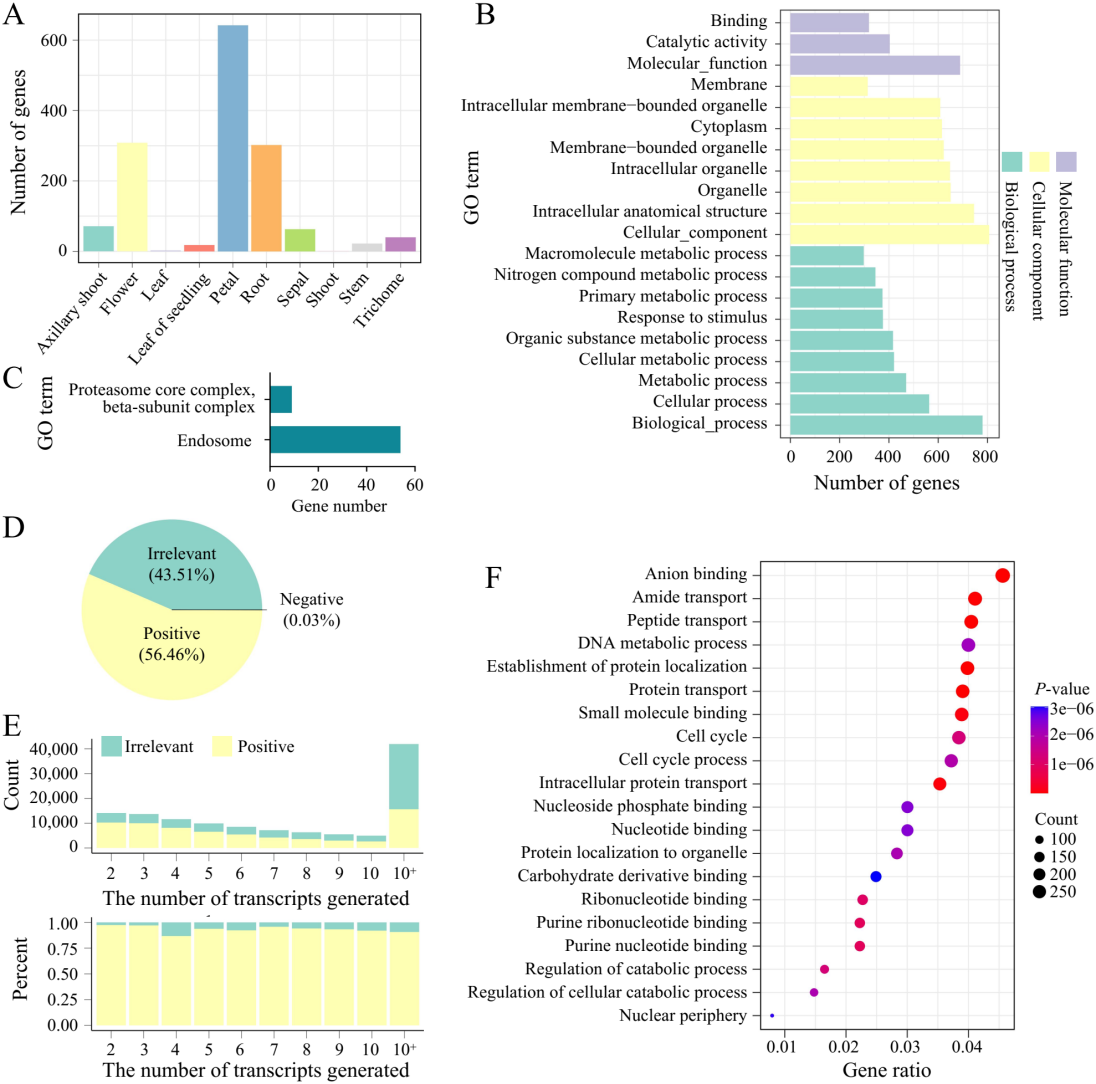
Transcript-host gene expression correlation and tissue-specific enrichment analysis. **(A)** The number of tissue-specific genes in different tissues. **(B)** GO enrichment analysis of tissue-specific genes in trichomes. **(C)** GO enrichment analysis of tissue-specific transcripts in trichomes. **(D)** Proportions of transcripts that are positively, negatively, or irrelevantly correlated with their host genes. **(E)** The count (top) and expression ratios (bottom) of transcripts that are positively correlated or irrelevant to their host genes. The "10+" on the x-axis represents genes that generated more than 10 transcripts. **(F)** GO enrichment analysis of transcripts that are irrelevant to the expression of their host genes.

To investigate the differences in expression patterns between transcripts and genes, we compared the number of tissue-specific transcripts and genes across different tissues, as well as the expression correlations between transcripts and their corresponding host genes. When comparing tissue-specific genes and transcripts, we found that the number of tissue-specific expressions was similarly distributed across tissues for both genes and transcripts. However, in trichomes, the number of tissue-specific transcripts far exceeded that of genes (**Figure 3A**). Trichomes are specialized epidermal cells in the aerial parts of plants and are associated with the plants response to biotic and abiotic stresses (Pattanaik et al., 2014). To explore the functions of the tissue-specific transcripts in trichomes, we analyzed the functions (GO terms) of the host genes of these transcripts and found that they are mainly involved in biological processes and cellular components, such as primary metabolic processes, nitrogen compound metabolic processes, and response to stimuli (**Figure 3B**). Furthermore, GO enrichment analysis revealed that these host genes are significantly enriched in endosome-related genes (**Figure 3C**). Endosomes are a group of heterogeneous organelles responsible for sorting and delivering internalized materials from the cell surface and transporting materials from the Golgi apparatus to lysosomes or vacuoles, playing crucial roles in plant hormone and defense signaling (Huotari and Helenius, 2011; Contento and Bassham, 2012). This suggests that the abundant transcripts generated by AS in trichomes may be an important mechanism by which plants respond to biotic and abiotic stresses. Enrichment analysis of host genes for tissue-specific transcripts across all tissues showed that tissues such as flowers, petals, and roots had the most enrichment results (**Supplementary Figure S1**, **Supplementary Table S3**). In the flower tissue, GO terms related to floral organ development were found, indicating that AS is also necessary for producing various transcripts to meet the growth requirements during flower morphogenesis.

Next, we calculated the expression correlations between transcripts and their host genes. A total of 130,567 transcript-gene pairs were analyzed, of which 56.44% (73,694 transcripts) showed a positive correlation with their host genes, while 43.5% (56797) of the transcripts were not correlated with their host genes (**Figure 3D**). As the number of transcript types per gene increased, the number of transcripts were irrelevant to gene expression also increased (**Figure 3E**). For instance, among the 54 genes enriched in the endosome GO term, 61.1% (294 transcripts) of the transcripts were not correlated with the expression of their host genes, which is significantly higher than the number of positively correlated transcripts. This suggests that tobacco may generate more transcripts through AS to adapt to external stresses. To explore the functions of these transcripts that are not correlated with their host genes, we conducted enrichment analysis, which revealed that these transcripts are mainly enriched in GO terms such as purine nucleotide binding, protein transport, and intracellular protein transport (**Figure 3F**). Genes related to purine nucleotide binding are essential for repairing certain DNA damage during plant vegetative growth and play roles in plant development and defense (Islam et al., 2020), while intracellular protein transport is also involved in various stress responses in plants (Kamal et al., 2020; Wolff et al., 2021). These findings further emphasize that transcript-level resolution is higher than gene-level resolution in transcriptome analysis, particularly in the study of stress-related genes.

### Analysis of ASE types

**Figure 4:**
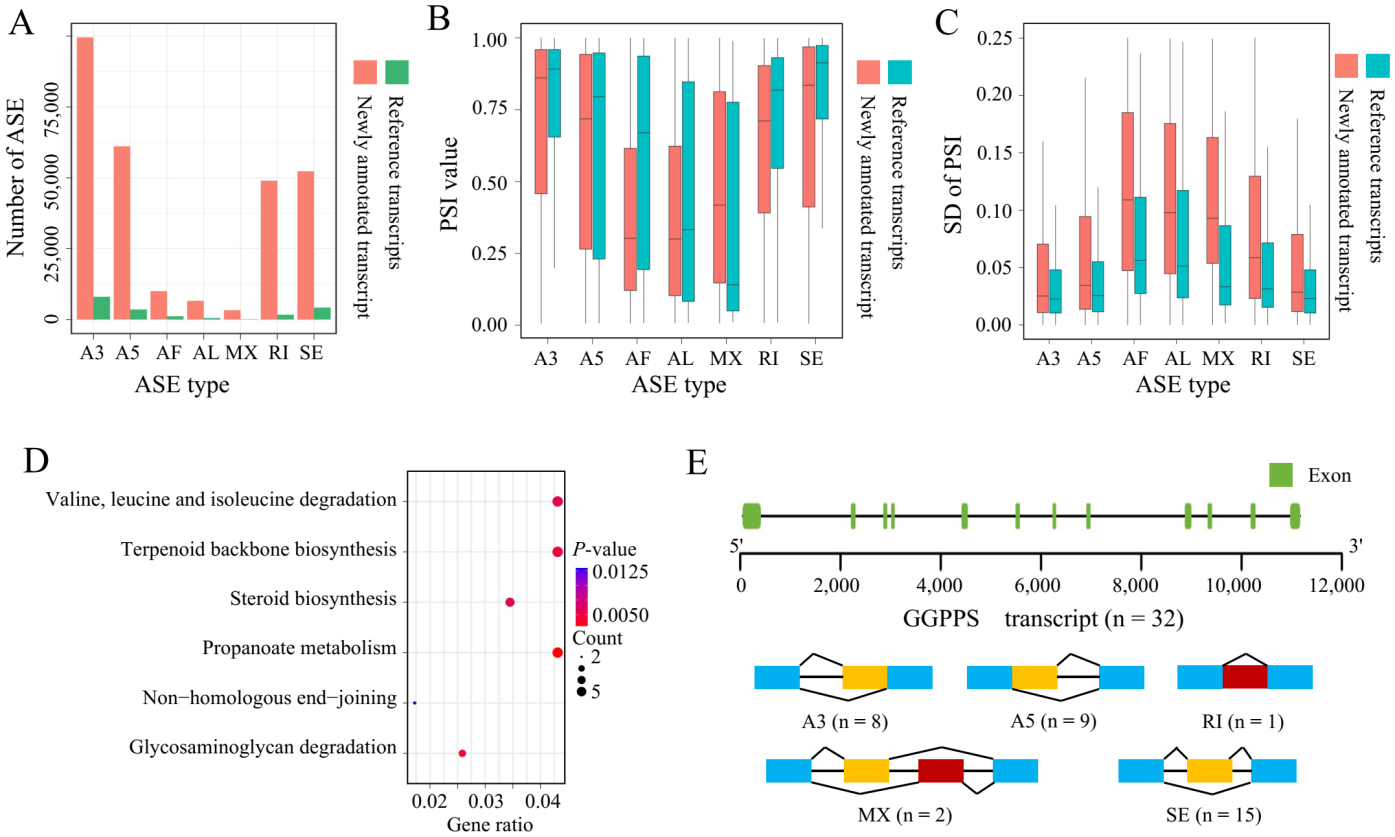
Analysis of AS types. **(A)** Comparison of different ASE types between transcripts annotated in the reference genome and newly annotated transcripts. **(B)** Relative expression levels (PSI) of different ASE types in transcripts annotated in the reference genome and newly annotated transcripts. **(C)** Variability in relative expression levels (PSI) of different ASE types between transcripts annotated in the reference genome and newly annotated transcripts. **(D)** KEGG enrichment analysis of the top 1,000 transcripts with the highest variance in PSI. **(E)** Schematic of splicing event types identified in the *GGPPS*.

To further investigate transcript diversity, we compared the transcripts annotated in the reference genome with newly annotated transcripts across different types of ASEs. We quantified levels of intron retention (RI), exon skipping (SE), alternative 3’-acceptor (A3), alternative 5’-donor (A5), alternative first exon (AF), alternative last exon (AL), and mutually exclusive exon (MX) splicing events. In this study, we identified a total of 107,140 ASEs occurring in 17,758 genes. Notably, A3 events accounted for the largest proportion of ASEs in both transcripts annotated in the reference genome and newly annotated transcripts (**Figure 4A**). Previous studies have reported similar results in tobacco using different tools to predict ASEs. However, it is well-established that RI are the most frequent type of AS event in plants, while SE dominate in humans (Wang and Brendel, 2006; Barbazuk et al., 2008; Filichkin et al., 2010; Zhou et al., 2024). Since the reference genome used in this study has only 64% of the genome anchored to pseudomolecules (Edwards et al., 2017), further identification of ASEs based on a higher-quality genome is required to determine whether this phenomenon is a species-specific characteristic of tobacco. Additionally, we used SUPPA2 to calculate the relative expression levels of different AS types, represented by “percent spliced in” (PSI). The expression levels of newly annotated transcripts in each splicing class did not show significant differences compared to those annotated in the reference genome. However, newly annotated transcripts in AF and AL classes exhibited lower relative expression levels compared to those in the reference genome (**Figure 4B**). The expression of newly annotated transcripts tended to be more variable, with isoforms produced by AF, AL, and MX showing greater variance among all splicing types (**Figure 4C**). Consequently, we conducted KEGG gene enrichment analysis on the top 1,000 transcripts with the greatest expression variance within the same ASE, finding that these transcripts were mainly enriched in pathways such as valine, leucine, and isoleucine degradation, terpenoid backbone biosynthesis, and steroid biosynthesis (**Figure 4D**). Terpenoids are the largest class of plant metabolites, playing crucial roles in essential plant processes such as respiration, photosynthesis, growth, development, reproduction, and environmental adaptation (Rodríguez-Concepción and Boronat, 2002; Gershenzon and Kreis, 2018). Our results showed that the host genes of these transcripts include genes involved in the terpenoid backbone biosynthesis pathway, such as geranyl diphosphate synthase (GGPPS) and farnesol kinase (FOLK). We analyzed the number and types of transcripts produced by GGPPS and found that GGPPS generated a total of 32 transcripts, encompassing 5 types of ASEs, indicating a high degree of transcript diversity. Geranyl diphosphate (GPP), synthesized by GGPPS, is the entry point for the synthesis of many monoterpenoid end products, and these monoterpenoids play important roles in plant-insect interactions by directly repelling or deterring herbivorous insects (Yin et al., 2017). Furthermore, we analyzed the proportion of tissue-specific expression among these 1,000 transcripts. We found that the highest number of tissue-specific transcripts were expressed in 10 tissues (blade, lamina, and midrib were combined with leaf), suggesting that the high variability of terpenoid biosynthesis-related transcripts in tobacco during polyploidization may be a key mechanism for responding to environmental changes (**Figure 4E**, **Supplementary Figure S2**).

### Regulatory impact of sQTLs on functional genes through ASEs

**Figure 5:**
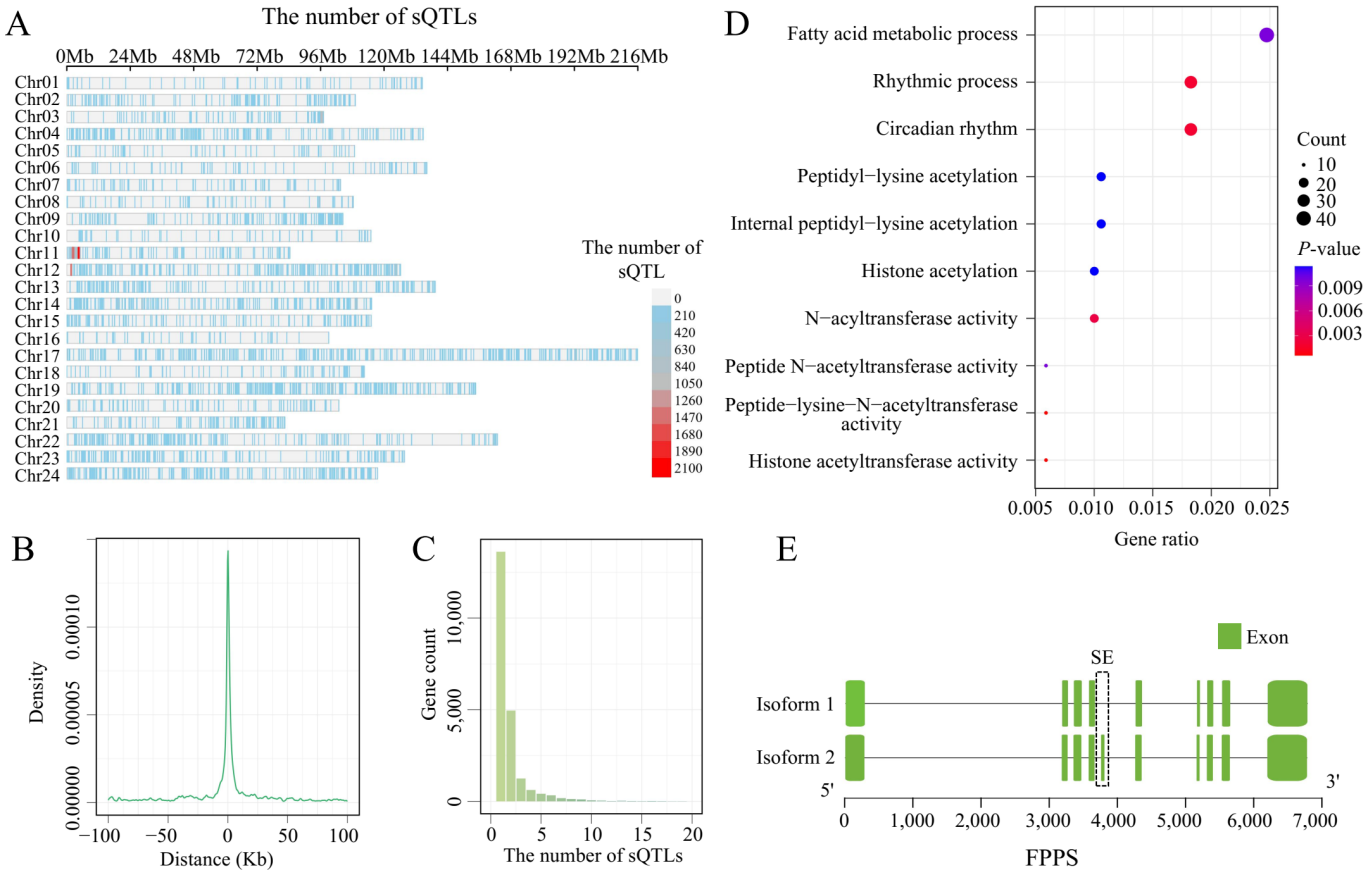
The identification of sQTLs in tobacco leaves. **(A)** The density distribution of sQTLs across chromosomes. **(B)** Position of sQTL variants in relation to the splice junction. **(C)** Statistics on the number of ASEs regulated by sQTLs. **(D)** GO enrichment analysis of the genes corresponding to ASEs regulated by sQTLs. **(E)** Schematic representation of the ASE occurring in the *FPPS* (*Nitab4.5_0000368g0370*).

Although this study has revealed the presence of widespread and diverse ASEs in tobacco, the regulatory mechanisms of these ASEs remain to be studied further. To analyze the impact of SNPs on ASEs, we used the PSI of 178,676 ASEs from 375 leaves as the phenotype and 598,293 SNPs as the genotype for sQTL analysis. There were 21,763 SNPs significantly associated with 9,068 ASEs with FDR<0.05 as the significant threshold (**Supplementary Table S4**. These SNPs significantly associated with ASEs are unevenly distributed on the chromosomes, with certain regions on chr11 and chr12 showing higher densities (**Figure 5A**). Comparing the distances between these SNPs and ASEs, we found that the number of cis-sVariants is inversely proportional to the distance from the ASE, with 80% of the cis-sVariants located within 10 kb of the ASE (**Figure 5B**). Most SNPs regulate only 1 to 2 ASEs, but a small number of SNPs regulate more than 10 ASEs. These SNPs may be key regulatory factors, exerting significant influence on gene expression regulation by affecting multiple ASEs simultaneously (**Figure 5C**). By mapping the ASE coordinates to genes, we identified sVariants that regulate genes through the modulation of ASEs in the sQTL analysis. A total of 3,086 genes were involved in sQTL (**Supplementary Table S5**), with an average of 2.4 genes regulated by each sVariant. Functional enrichment analysis of these genes revealed that these sGenes are mainly enriched in histone modification-related terms, such as histone acetyltransferase activity, peptide-lysine-N-acetyltransferase activity, and N-acyltransferase activity (**Figure 5D**). These results suggest that these sQTLs may influence chromatin structure and gene expression through histone acetylation and other modifications, further contributing to epigenetic regulation. Additionally, a large number of sGenes were annotated to resistance-related pathways in the KEGG database, including metabolic pathways (372 genes), plant hormone signal transduction (33 genes), and terpenoid backbone biosynthesis (7 genes). Although these pathways did not reach significant enrichment levels in the enrichment analysis, their associations suggest that these sQTLs may have potential importance in plant resistance responses. For example, in the terpenoid backbone biosynthesis pathway, the gene *farnesyl diphosphate synthase* (*FPPS*, Nitab4.5_*0005478g0020*) has one ASE, and this ASE is significantly regulated by two sQTLs (**Figure 5E**). FPPS and GGPP are two major isoprenoid intermediates. Similar to GGPP, FPP is mainly synthesized by FPPS, playing an important role in the terpenoid compound pathway, which is crucial in plant secondary metabolism and stress response (Ueoka et al., 2020; Conart et al., 2023). These results suggest that sQTLs may regulate FPPS through ASEs, potentially leading to changes in FPP expression levels, which could directly affect the synthesis and accumulation of terpenoid compounds, thereby influencing plant resistance. These findings not only enrich our understanding of tobacco gene regulatory mechanisms but also provide new insights into the intricate structure of gene regulatory networks.

## Discussion

This study analyzed transcript expression in 599 RNA-seq samples from 13 different tobacco tissues, creating the first transcript expression atlas for tobacco. Compared to the 35,519 transcripts annotated from protein-coding genes in the reference genome, this research identified a total of 149,072 transcripts derived from protein-coding genes. Notably, 76% of these are novel transcripts, originating from 70% of the genes, indicating the widespread occurrence of AS in tobacco. While these transcripts may include potential false positives, such as those with non-canonical splice junctions, fragmented transcripts, or redundant transcripts, this study provides a valuable resource for molecular biologists and breeders as a reference for future research and applications. AS is a crucial post-transcriptional regulatory process in which a single gene can produce multiple transcripts, thereby increasing the complexity of the transcriptome, gene regulation, and proteome diversity in multicellular eukaryotes (Mazzucotelli et al., 2008; Chaudhary et al., 2019). In plants, 60-70% of intron-containing genes undergo AS (Pan et al., 2008; Marquez et al., 2012). For example, in the potato genome, an important economic crop, approximately 33,000 genes have been identified (Phytozome v.13), with over 44,000 predicted transcripts, and more than 7,000 genes are known to produce multiple transcripts (Betz et al., 2024). The proportion of multi-transcript genes in the potato genome is much lower than what we observed in tobacco. However, this does not imply that potato genes do not generate a large number of transcripts. Rather, it highlights the effectiveness of transcript expression analysis on large and diverse RNA-seq datasets to uncover the AS patterns of a species like this study.

Polyploid plants often exhibit an increase in genome size, which can lead to enhanced hybrid vigor and improved stress resistance compared to their ancestors. This may influence the size of the transcriptome or the abundance of transcripts (Yang et al., 2014; Han et al., 2016; Song et al., 2020). The novel transcripts annotated in this study could be a result of tetraploid tobacco adapting to environmental changes and enhancing its immune responses. Similar observations have been made in other polyploid plants. For instance, Li et al. reported that in the allotetraploid rapeseed, there is an increase in the number of splicing isoforms, pre-mRNAs undergoing ASE, pre-mRNAs undergoing APA, and transcription factors (TFs) (Li et al., 2022). Yu et al. also found that polyploidization in wheat led to widespread ASE (Yu et al., 2020). Additionally, some studies suggest that genes may exhibit changes in transcript proportions in response to biotic interactions without altering overall mRNA expression levels (Rigo et al., 2019). Therefore, investigating the tissue-specific transcripts could offer new insights into the evolution, environmental adaptation, and immune responses of tetraploid tobacco. Specifically, our study reveals extensive tissue-specific expression of tobacco transcripts, with notable differences in expression patterns between transcripts and genes. For example, in key functional organs or tissues, trichome-specific transcripts account for 39% of the total, whereas trichome-specific genes constitute only 2.8% of the total. Trichomes are considered the first line of defense in plants, serving as physical and chemical barriers against herbivores and pathogens (Weinhold and Baldwin, 2011). They possess the ability to secrete and store a variety of secondary metabolites, such as terpenoids, phenylpropanoids, and acyl sugars. The presence of trichome-specific promoters and other regulatory elements drives the tissue-specific genes involved in secondary metabolite biosynthesis, leading to the accumulation of these metabolites (Wang et al., 2002). In this study, we identified a large number of trichome-specific transcripts in tobacco, particularly those derived from 92 genes related to secondary metabolites (https://www.genome.jp/pathway/map01110), providing a foundation for further research into the ecological role of trichomes. These results suggest that the changes in AS within specific tissues may be potentially linked to the environmental adaptability of tetraploid tobacco.

AS plays a crucial role in plant adaptation to abiotic stress and environmental constraints (Dubrovina et al., 2013; Staiger and Brown, 2013). For instance, Filichkin et al. discovered that temperature stress can alter transcript abundance and splicing patterns, thereby affecting both transcript and gene expression (Filichkin et al., 2010). In our study, the top 1,000 transcripts with the most significant changes in expression were enriched in pathways related to stress response and metabolism, such as the Terpenoid backbone biosynthesis pathway. Among these, the transcripts produced by the tobacco GGPPS gene exhibited particularly high variability in expression. GGPPS, located in plastids, can interact with GPPS to modify product specificity, thereby efficiently producing GPP. To prevent this process from becoming uncontrolled, plants typically regulate it at the transcriptional level (Tholl et al., 2004; Pateraki and Kanellis, 2008; Wang and Dixon, 2009). Our findings show that GGPPS generates a total of 32 transcripts through 35 ASEs across 5 different splicing types, suggesting that the generation of transcript diversity and differential expression via ASEs may be a key mechanism for the regulation of the GGPPS gene in tobacco.

AS is regulated through both co-transcriptional and post-transcriptional mechanisms, with sQTL explaining part of the variation in transcript isoform ratios (Jia et al., 2020; Li et al., 2020; Wilkinson et al., 2020). In this study, 3,086 sQTL-regulated genes were identified in tobacco leaves, with significant enrichment in terms such as histone acetyltransferase activity, reflecting the complexity of regulatory mechanisms. Previous research revealed a dynamic interplay between DNA methylation, histone modifications, and AS. For example, pre-messenger RNA can precisely regulate AS by recruiting histone-modifying enzymes (Zhou et al., 2014). Histone acetyltransferases (HATs), as key epigenetic regulators, transfer acetyl groups to lysine residues on histones, leading to histone acetylation—a process directly linked to chromatin remodeling and the regulation of gene transcription (Roth et al., 2001). Although we cannot yet assess the direct impact of sQTLs on histone acetylation, exploring the potential connections between these sQTL-regulated genes and histone acetylation could advance our understanding of the regulatory mechanisms underlying gene expression in tobacco.

We also identified 7 sQTL-regulated genes within the terpenoid backbone biosynthesis pathway, which undergo 9 ASEs and are influenced by 19 sQTLs. Terpenoid biosynthesis plays a crucial role in plant secondary metabolism, with many secondary metabolites involved in the plants response to biotic and abiotic stresses (Nishida, 2014). The synthesis of these metabolites is regulated at multiple molecular levels, including AS. For instance, in rice, the WRKY transcription factor regulates diterpenoid biosynthesis through AS, contributing to the plant defense response (Liu et al., 2016). The research of AS in relation to these secondary metabolites enhances our understanding of its regulatory role in plant metabolism and offers insights for crop improvement. In our study, we found that the FPPS, involved in tobacco terpenoid synthesis, contains 1 ASE that is significantly regulated by two sQTLs. This suggests that sQTLs may play a role in regulating the synthesis and accumulation of terpenoid compounds. Overall, this research provides a comprehensive view of the transcriptomic features of polyploid tobacco. Notably, the high transcript diversity in tobacco may significantly impact the production of secondary metabolites and the response to biotic and abiotic stresses of plants, offering new insights into the transcriptional regulation of tobacco.

## Data Availability

The original contributions presented in the study are included in the article/**Supplementary Material**. Further inquiries can be directed to the corresponding author/s.

## Supplementary material

Supplementary Figure 1 GO enrichment results of tissue-specific transcripts.

Supplementary Figure 2 Transcripts of GGPPS.
