# Supplementary figures and images for: A gene expression atlas of Nicotiana tabacum across various tissues at transcript resolution
Source: Front Plant Sci. 2025 Feb 7;16:1500654. doi: 10.3389/fpls.2025.1500654 (PMC11841470; doi:10.3389/fpls.2025.1500654)

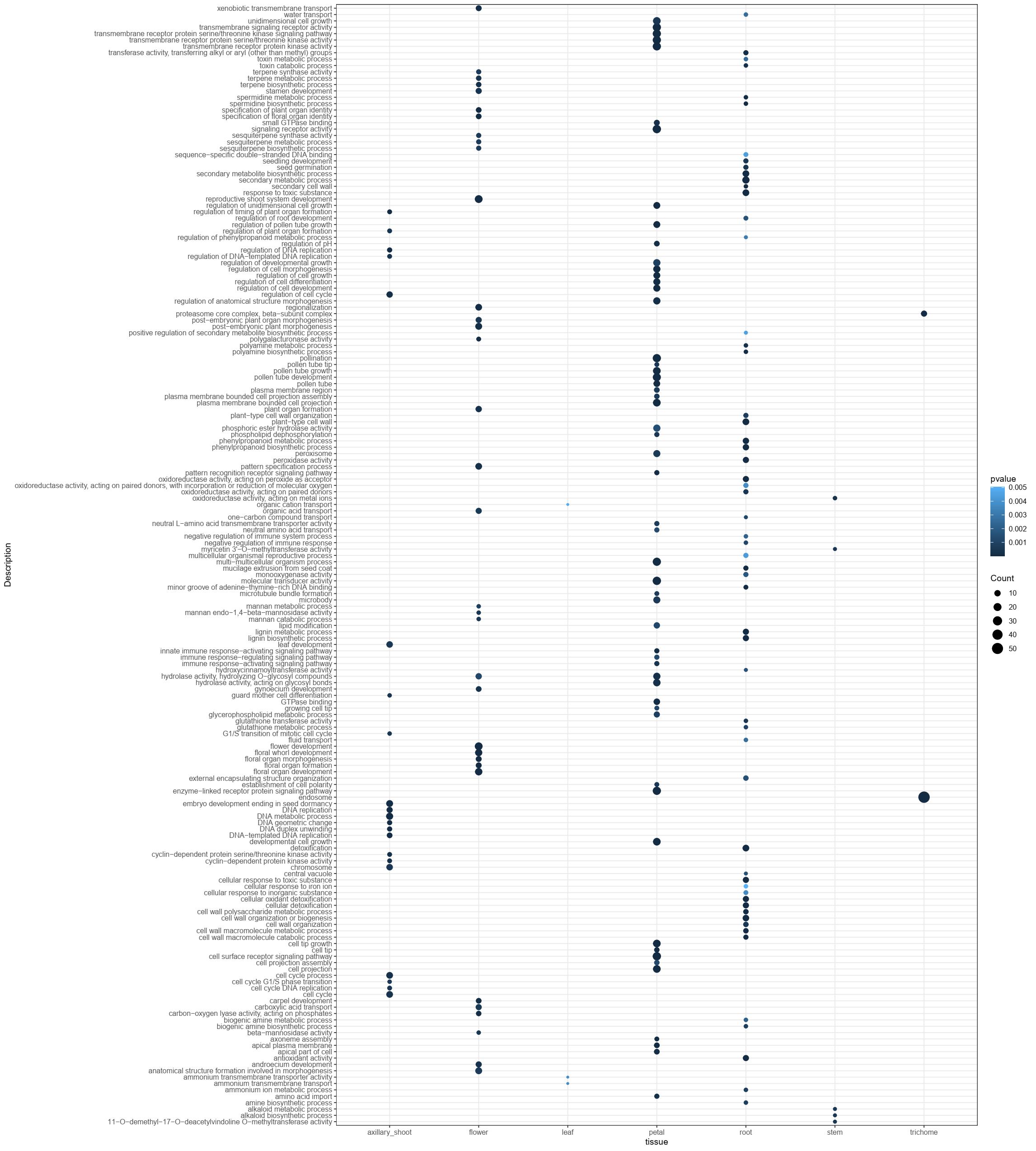

Supplement: Supplementary Figure 1 — GO enrichment results of tissue-specific transcripts. [file Image1.jpeg]

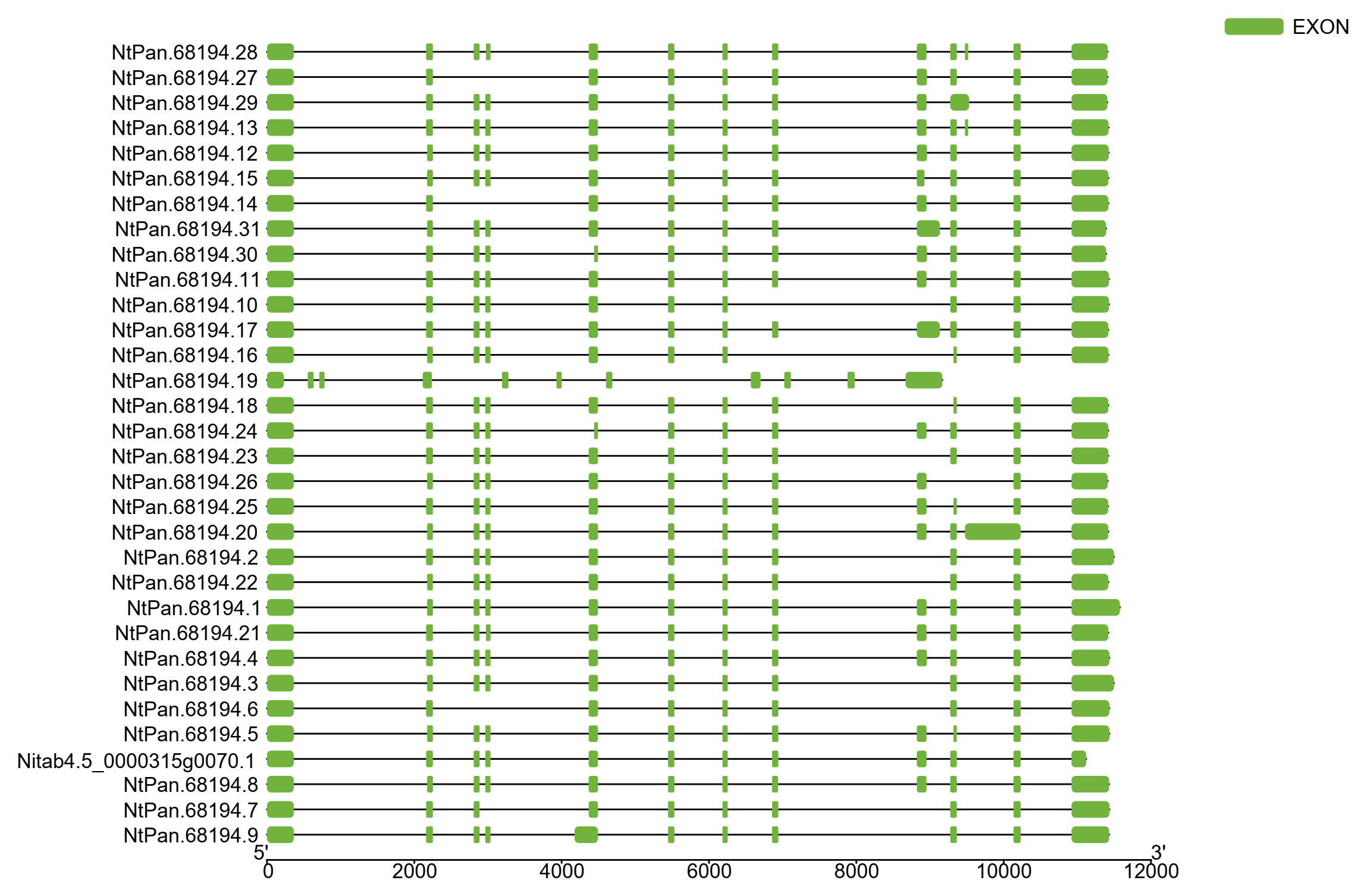

Supplement: Supplementary Figure 2 — Transcripts of GGPPS. [file Image2.jpeg]
